# Decoding Immunodeficiencies with Artificial Intelligence: A New Era of Precision Medicine

**DOI:** 10.3390/biomedicines13081836

**Published:** 2025-07-28

**Authors:** Raffaele Sciaccotta, Paola Barone, Giuseppe Murdaca, Manlio Fazio, Fabio Stagno, Sebastiano Gangemi, Sara Genovese, Alessandro Allegra

**Affiliations:** 1 Hematology Unit, Department of Human Pathology in Adulthood and Childhood “Gaetano Barresi”, University of Messina, Via Consolare Valeria, 98125 Messina, Italy; sciaccottaraffaele@gmail.com (R.S.); baronepaola2903@gmail.com (P.B.); manliofazio@hotmail.it (M.F.); fabio.stagno@unime.it (F.S.); aallegra@unime.it (A.A.); 2 Department of Internal Medicine, University of Genova, 16126 Genova, Italy; 3 Allergology and Clinical Immunology, San Bartolomeo Hospital, 19038 Sarzana, Italy; 4 Allergy and Clinical Immunology Unit, Department of Clinical and Experimental Medicine, University of Messina, Via Consolare Valeria, 98125 Messina, Italy; gangemis@unime.it; 5 Institute for Biomedical Research and Innovation (IRIB), National Research Council of Italy (CNR), 98164 Messina, Italy; sara.genovese@irib.cnr.it

**Keywords:** primary immunodeficiency, secondary immunodeficiency, artificial intelligence, immunopeptidomic profiling, genetic mutation, immune system, machine learning, deep learning, biomarker, vaccines

## Abstract

Primary and secondary immunodeficiencies comprise a wide array of illnesses marked by immune system abnormalities, resulting in heightened vulnerability to infections, autoimmunity, and cancers. Notwithstanding progress in diagnostic instruments and an enhanced comprehension of the underlying pathophysiology, delayed diagnosis and underreporting persist as considerable obstacles. The implementation of artificial intelligence into clinical practice has surfaced as a viable method to enhance early detection, risk assessment, and management of immunodeficiencies. Recent advancements illustrate how artificial intelligence-driven models, such as predictive algorithms, electronic phenotyping, and automated flow cytometry analysis, might enable early diagnosis, minimize diagnostic delays, and enhance personalized treatment methods. Furthermore, artificial intelligence-driven immunopeptidomics and phenotypic categorization are enhancing vaccine development and biomarker identification. Successful implementation necessitates overcoming problems associated with data standardization, model validation, and ethical issues. Future advancements will necessitate a multidisciplinary partnership among physicians, data scientists, and governments to effectively use the revolutionary capabilities of artificial intelligence, therefore ushering in an age of precision medicine in immunodeficiencies.

## 1. Immunodeficiency’s Two Faces: Primary and Secondary

The immune system is a complex system of cells, tissues, and organs that collaborate to protect the body from external agents that may be detrimental, including bacteria, viruses, fungi, parasites, and toxins. Impairments in any elements of the immune system result in immunodeficiency, classified as primary or secondary based on its underlying causes [1,2]. Primary immunodeficiency disorders (PIDDs) or primary immunodeficiencies (PIs) are inherited abnormalities of the immune system that result in heightened vulnerability to infections and/or immunological dysregulation. They frequently involve genetic mutations in immune system components and encompass antibody deficit, complement deficiency, phagocytic insufficiency, and combined immunological deficiency, among others [3,4,5]. PIDDs are expected to manifest in approximately 1 in 1000 to 2000 live births, with significant associated morbidity and mortality rates [6]. Despite the overall low frequency of PIDD, estimated at 50.5 per 100,000, a recent study identified a consistent increase in prevalence from 2001 to 2007 [7]. A group of over 400 disorders known as inborn errors of immunity (IEI) are defined by deficiencies in the immune system’s function and/or development, predominantly impacting the juvenile demographic [8,9]. Regardless of the advancements in the characterization of PIDD/IEIs, diagnostic rates beyond the neonatal phase remain unchanged [10]. IEIs are frequently underdiagnosed and underreported conditions. A total of 70 to 90 percent of individuals with IEI remain undiagnosed, even in nations with established IEI screening and care initiatives [11]. Diagnostic delays exacerbate morbidity in individuals with PIDD/IEI, inflate medical costs, and create a burdensome diagnostic journey that poses challenges for both patients and clinicians [12]. Nonetheless, certain disorders lack genetic foundations, potentially compromising the integrity of the immune system and resulting in secondary immunodeficiencies. The most common causes include recognized conditions such as HIV infection; neoplastic diseases, particularly hematological ones like multiple myeloma (MM), chronic lymphocytic leukemia (CLL), or myelodysplastic syndromes (MDS); as well as chronic illnesses such as diabetes or renal failure [13]. A study highlighted how 80% of patients with CLL suffer from infections, which remain the leading cause of mortality among these patients [14] (Table 1). Secondary immunodeficiencies (SID) are more common among the adult population, and they can simultaneously impact both innate and adaptive immunological responses, as well as various components of the immune system [15]. These conditions can be transient and thus reversible; nonetheless, there are instances in which the defect persists, with important consequences in terms of comorbidity [16,17]. In recent years, there seems to be a tendency toward an increase in these secondary forms, and the contributing factors include population growth, the lengthening of the average age, increasing prevalence of malignancies, and the use of immunosuppressive agents (such as biologics and chimeric antigen receptor [CAR] T-cell therapy) and the diagnostic progresses made for detecting those conditions linked to SID, such as CLL, which saw a 26% increase in diagnoses between 2005 and 2015 [16,18]. It is important to recognize and treat SID promptly to avoid complications that can lead to morbidity and mortality.

### Artificial Intelligence: Revolutionizing the Medical Field

The use of artificial intelligence (AI), augmented intelligence, and machine learning (ML) is becoming increasingly widespread in the healthcare industry. These technologies provide assistance to medical professionals in the analysis of complex datasets, improve the diagnosis of illnesses, and facilitate clinical decision making [19]. AI empowers computers to replicate human cognitive functions through observation, problem-solving, and learning, while ML and its subset, deep learning (DL), pertain to the capacity of systems to learn, identify patterns, and enhance performance progressively [20]. Augmented intelligence utilizes machine learning to facilitate the actionable use of data, as opposed to the autonomous substitution of human cognition. Artificial neural networks (ANNs) are a category of deep learning models that employ several layers to process data inputs for predictive or regression tasks using a nodal architecture that simulates the functioning of the human brain [21]. An increasing number of research settings have adopted the use of DL algorithms like these to analyze complicated and high-dimensional data. The mechanics of AI focus on the examination of machine-readable components organized to anticipate a certain outcome, such as categorization or diagnosis [22]. The application of AI algorithms within the healthcare sector is contingent upon the development of validated datasets that are sourced from both structured and unstructured data of significance [23]. Healthcare datasets, including electronic health records (EHRs) and pathology images, such as eosinophils in biopsies of eosinophilic intestinal disorders, pose distinct challenges and opportunities for data extraction [24]. A primary feature of AI in healthcare is automated illness diagnosis, wherein electronic phenotyping (EP) of patients exhibiting certain clinical characteristics facilitates machine-readable data and identification [25,26]. The establishment of accurate EPs has demonstrated efficacy in the exploration and identification of extensive datasets to recognize disease entities [27]. AI devices can be broadly classified into two principal categories. The first type encompasses ML techniques that scrutinize structured data, which include imaging, genetic, and electrophysiological information. ML processes in healthcare settings aim to group patients’ characteristics (age, gender, disease history, etc.) or predict the likelihood of illness consequences [28]. ML algorithms can be categorized into two primary types based on the incorporation of outcomes: unsupervised learning and supervised learning. Unsupervised learning is recognized for its capability in extracting features, whereas supervised learning is appropriate for predictive modeling by establishing relationships between patient characteristics (input) and the outcome of interest (output). In recent times, semi-supervised learning has emerged as an integrated approach that combines elements of both unsupervised and supervised learning, making it applicable in situations where outcomes are unavailable for specific subjects. Clustering and principal component analysis represent two significant approaches within the realm of unsupervised learning, grouping subjects with similar characteristics into clusters, independent of the outcome information. Clustering methods produce patient cluster labels by maximizing and minimizing patient similarity within and across clusters. Popular clustering algorithms (PCA) such as k-means clustering, hierarchical clustering, and Gaussian mixture clustering are common examples of popular algorithms for clustering. PCA is mostly used for dimension reduction, particularly when a characteristic is measured across a high number of parameters, like the number of genes in genome-wide association research. PCA maps the data to a few principal component (PC) directions without sacrificing too much information about the topics. Sometimes, PCA can be used first to reduce the dimension of the data, followed by clustering to group the patients. On the other hand, supervised learning takes into account the subjects’ outcomes as well as their characteristics and goes through a specific training procedure to discover the optimal outputs linked with the inputs that are closest to the outcomes on average. Supervised learning yields more therapeutically pertinent outcomes than unsupervised learning; hence, AI applications in healthcare predominantly utilize supervised learning. The image, EP, and genetic data are machine-readable, allowing for the direct application of ML algorithms following appropriate preprocessing or quality control measures. Nonetheless, substantial amounts of clinical data exist as narrative text, including physical examinations, laboratory findings, operation notes, and discharge summaries, which are unstructured and unintelligible to computer programs. In this setting, Natural Language Processing (NLP) aims to extract pertinent data from narrative text to support clinical decisions [29]. The second group includes NLP techniques that derive information from unstructured data, such as clinical notes and medical journals, to enhance and augment organized medical data. The NLP processes aim to convert texts into machine-readable structured data, which may subsequently be analyzed using ML approaches [30]. An NLP pipeline consists of two primary elements: (1) text processing and (2) classification. Utilizing text processing techniques, the NLP system detects a range of keywords pertinent to diseases within the clinical states, drawing from historical databases [31]. Following this, a subset of the keywords is chosen by analyzing the influence that they have on the categorization of the normal and abnormal conditions. In order to facilitate clinical decision making, the verified keywords are subsequently included into and enhanced the structured data. NLP pipelines have been created to support healthcare decisions by advising therapy systems, evaluating adverse reactions, and similar tasks.

## 2. Early Immunodeficiency Diagnosis and AI

### 2.1. General Considerations

**Figure 1:**
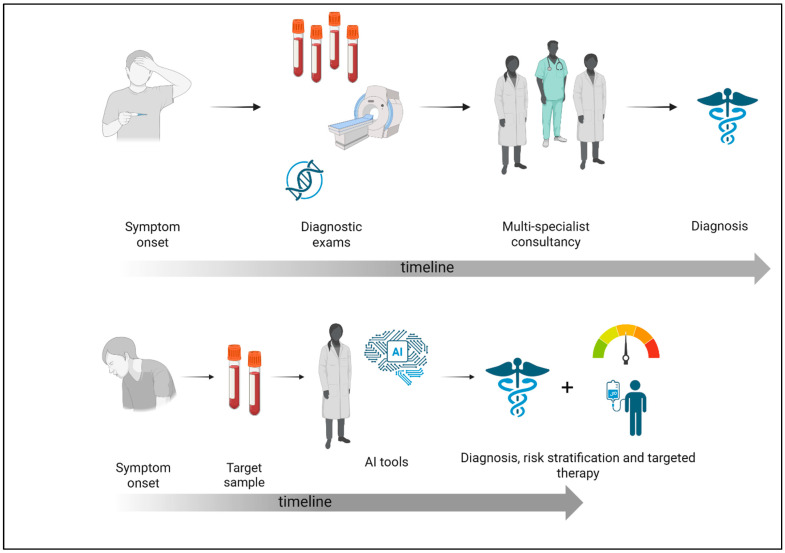
Comparison between the traditional diagnostic process and the AI-supported approach in immunodeficiency. This figure shows the impact of artificial intelligence on the diagnostic pathway for immunodeficiencies. Traditionally, diagnosis predominantly depends on physician-led evaluations, frequently necessitating numerous appointments, comprehensive differential diagnoses, and sequential testing, resulting in considerable delays. Conversely, AI-enhanced systems examine extensive patient data to discern trends and risk profiles indicative of immunodeficiencies (created with Biorender.com, accessed on 15 May 2025).

Despite an improvement in diagnostic tools, IEIs remain a major challenge, with often long delays in detecting these conditions [32]. Due to their diverse and frequently overlapping clinical symptoms, many illnesses are difficult to diagnose, which highlights the necessity for a new, personalized approach [33]. Recent studies have utilized data collected from EHR to create scores that indicate the likelihood of primary PIDD, assisting clinicians in early diagnosis. Advanced machine learning algorithms can identify patterns in EHR data, facilitating predictions regarding patient diagnosis and outcomes. Through the integration of enormous volumes of associated data from various sources, these algorithms facilitate early detection and support clinical decisions for diagnosis and treatment. The use of EHRs rather than International Classification of Diseases (ICD) codes can provide useful information for early diagnosis of PIDD. Patients with PIDD often receive symptomatic treatments rather than interventions targeting the underlying cause. The documented treatments within the healthcare system reveal patterns that may enhance the detection of PIDD. In fact, using a machine learning framework to incorporate variables other than diagnosis codes, such as past symptoms and treatment, could favor an early diagnosis [34] (Figure 1).

It is increasingly recognized that the integration of AI into laboratory methodologies, particularly multiparameter flow cytometry (MFC), offers substantial advancements in the diagnosis of PIDs [35]. While conventional MFC remains indispensable for characterizing immune cell subsets and identifying hallmark abnormalities, traditional manual gating approaches are constrained by operator-dependent variability, time demands, and their inability to fully capture the complexity of high-dimensional datasets [36]. AI-driven analytical platforms overcome these challenges by automating essential processes such as data quality control, normalization, visualization, cell population identification, and diagnostic classification, thereby enhancing processing speed and ensuring consistent, reproducible results independent of subjective interpretation [37]. Beyond flow cytometry, AI also serves as a powerful tool for the detection and classification of cellular phenotypes: AI-powered image-based analyses surpass traditional microscopy by rapidly processing extensive datasets and identifying subtle phenotypic alterations that might otherwise go unnoticed. Moreover, in immunology, ML has notably advanced the prediction of the immunopeptidome, supporting the development of personalized vaccines [38]. Although mass spectrometry remains the gold standard for mapping the immunopeptidome, ML models provide a faster and more cost-effective alternative by accurately predicting Major Histocompatibility Complex (MHC)-I and MHC-II epitopes and aiding in the design of adjuvants and immunomodulatory sequences [39]. Collectively, these AI-driven approaches are transforming laboratory diagnostics and immunological research, with significant implications for improving the management and treatment of patients with immunodeficiencies.

### 2.2. Warning Signs to AI-Powered Diagnosis: IEI Identification’s New Era

Efforts to enhance the early identification of IEIs have a longstanding history. In 1993, the Jeffrey Modell Foundation established the 10 Warning Signs, marking one of the initial attempts to formalize the diagnoses indicative of potential immune deficiencies. The warning signs indicated recurrent infections as a significant theme, leading to a substantial rise in referrals to immunologists [40]. Nonetheless, the advocacy for these indicators as predictors of IEIs has been challenged, primarily due to their emphasis on infections rather than the immune dysregulation and syndromic features, as the European Society for Immunodeficiencies claims [41]. Charlotte Cunningham-Rundles and colleagues were among the initial groups to provide improvements to the warning signs derived from extensive health insurance claims databases [42]. By assigning weights to 350 ICD codes that matched the 10 Warning Signs in an extensive claims database, the Modell Foundation presented its Software for Primary Immunodeficiency Recognition, Intervention, and Tracking (SPIRIT) analyzer [43]. The initial step typically involves mapping segments of the medical record and standardizing them to terms compatible with software applications. Researchers at Vanderbilt University have developed a mapping of billing codes (ICD codes) and a standardized tool known as Human Phenotype Ontology (HPO) to translate ICD terms into phecodes [44]. Phecodes represent a simplified categorization of diagnoses relative to ICD codes, facilitating more efficient identification of phenotypes for computational purposes. This mapping facilitates the representation of phenotype syndromes modeled on Mendelian diseases through the utilization of clinical phenotypes obtained from electronic health records (EHR). Alternative groups have employed manual methods to capture phenotypes. In an effort to facilitate the diagnosis of Common Variable Immunodeficiency (CVID), Dr. Luiza Campos and the INTREPID team at University College London developed a phenotype capture tool, available in the United Kingdom, to compile a list of phenotypes of patients affected by an IEI. The tool utilized the HPO to catalog the phenotypic features of 886 identified patients with IEIs, resulting in the acquisition of 600 patients for the diagnosis of CVID. During the training of an algorithm, numeric weights are assigned to the many phenotypic features of a patient to reflect their significance in contributing to an IEI diagnosis. Another approach is based on the development of a machine learning-based tool that utilizes an expert panel to assign weights to 83 diagnosis codes, antibiotic usage, atypical immunoglobulin levels, and recurrent visits to primary care. In this work, the algorithm was applied to a cohort of 60,000 individuals aged 12 to 70 years to identify those at high risk, who were subsequently referred to an immunologist, with an estimated diagnostic delay decreased by 3 years [45]. Another significant example involves the application of a regression model to aid in the diagnosis of CVID. Professors Manish Butte and Bogdan Pasaniuc from the University of California developed a regression model utilizing data from approximately 190 patients with CVID and appropriate controls. The model incorporated 44 phecodes along with IgG levels to calculate a risk score for each patient. This method can identify patients with CVID with an advantage of up to 4 years. Their methodology has expanded to include the identification of undiagnosed patients within the five hospitals of the University of California health system. Sixty percent of undiagnosed patients recognized by the algorithm were characterized in a blinded chart review as “likely to have CVID”. Collectively, these recent initiatives have advanced beyond the 10 Warning Signs and can effectively identify patients with IEIs. Most training data for current efforts primarily concentrate on infections; however, these initiatives have also validated the significance of examining autoimmunity and inflammatory characteristics, including constitutional effects like failure to thrive [46]. An Italian study has challenged the prevailing notion that IEIs are precursors to cancer, instead suggesting that cancers may indicate underlying immune deficiencies. In fact, authors presented new indicators of immune dysregulation and IEIs: cancer, cytopenia, lymphoproliferative disorders, and myelodysplastic disorders [47]. A recent study demonstrates that information within the EHR related to differential diagnoses and treatment of symptoms related to PIDD can be utilized to identify PIDD. This study demonstrates that frequent use of specific components of the healthcare system may indicate the presence of PIDD. The authors demonstrate that incorporating laboratory tests, radiological orders, and medications from a patient’s historical interactions with a hospital enhances the detection of PIDD compared to relying solely on ICD-based comorbidities, resulting in improved discrimination, sensitivity, and specificity. The authors performed a retrospective study on patients with PIDD, utilizing inclusion criteria that encompassed PIDD-related diagnoses, immunodeficiency-specific medications, and low immunoglobulin levels. This study utilized machine learning models including logistic regression (LR), elastic nets (EN), and random forests (RF). Logistic regression is a statistical approach that posits a linear relationship between the log odds of the outcome and the predictors. EN is a model that imposes penalties on coefficients to mitigate crossover among predictors. RF models utilize a tree-based methodology that constructs multiple uncorrelated decision trees through random sampling of data. The ultimate prediction is derived by averaging the outputs from the decision trees. The score produced by this model serves to estimate the probability of PIDD, potentially minimizing diagnostic delays. This model, despite its limitations, may serve as a crucial initial step in developing an EHR alert for experts to consider a diagnosis of PIDD, thereby expediting treatment with immunoglobulin replacement therapy and potentially reducing the risk of morbidity and mortality [34]. Another study proposed that large language models (LLMs) could assist clinical immunologists by providing valuable recommendations for the diagnosis and management of PIs [48]. The utilization of LLMs as chatbots for clinical decision support presents a significant opportunity to enhance the quality of healthcare [49]. Consequently, extensive utilization of LLMs capable of delivering recommendations may enhance disease recognition. It is crucial to assess the effectiveness of current LLMs in delivering useful clinical recommendations regarding PIs. To evaluate this concept, the authors examined the responses of six LLMs regarding their capacity to deliver helpful information on 25 instances of PI. The clinical courses of patients with primary immunodeficiency were presented by five expert clinical immunologists to investigate the utility of large language models across the International Union of Immunological Societies spectrum and within the complexity of primary immunodeficiency [50]. Larger models typically demonstrate enhanced adaptability in downstream applications, especially in specialized or complex domain-specific natural language processing tasks. High-performing LLMs generally identified the correct diagnosis early or presented it as a strong candidate within the differential diagnosis [48] (Table 2).

### 2.3. AI-Enhanced Flow Cytometry: Early PID Detection Precision Tools

It is well acknowledged that multiparameter flow cytometry (MFC) is among the diagnostic technologies that are utilized the most frequently in the field of hematopathology. By staining cells with fluorescent antibodies, this high-dimensional method enables accurate detection and measurement of antigen expression [35]. The clinical suspicion of PID often emerges from a comprehensive evaluation of the patient’s medical history, emphasizing recurring infections, autoimmune diseases, and associated complications, due to the diverse and occasionally overlapping presentations of the condition. While genetic analysis is necessary for a clear evaluation, it may be costly and laborious. However, flow cytometry can detect distinct immune cell abnormalities in main PIs types [36]. For example, X-linked agammaglobulinemia (XLA) is defined by the lack of circulating B-cells and a significant decrease in serum immunoglobulins resulting from the Bruton’s tyrosine kinase (BTK) gene mutation, which can be identified through the absence of B-cells in peripheral blood. Additionally, flow cytometry can be utilized to identify XLA carriers through the analysis of monocytes [51]. Hepatosplenomegaly, autoimmune cytopenia, lymphadenopathy, and a higher incidence of lymphomas are the hallmarks of the uncommon hereditary condition of lymphocyte apoptosis known as autoimmune lymphoproliferative syndrome (ALPS) [52]. A characteristic of ALPS is the presence of CD4/CD8 double-negative T-cells (DNTs) expressing T-cell receptor αβ. Crucial diagnostic requirements for ALPS include TCRαβ+/DNT > 1.5% of total lymphocytes or >2.5% of CD3+ cells [53]. Flow cytometry allows for easy identification of the DNTs population. DiGeorge syndrome is a congenital immune deficiency disorder characterized by unusual T-cell development and dysfunction, resulting in a spectrum of atypical lymphoid subset levels, either high or low [54]. The defining characteristic is a notable reduction or absence of CD45RA+ T-cells and a decrease in switched memory B-cells, leading to impaired immune function, increased susceptibility to infections, and lowered immunoglobulin levels. Molecular and genetic testing is the definitive method used for diagnosing primary immunodeficiencies (PIs); however, flow cytometry has emerged as an essential tool for identifying and evaluating the immunologic function in affected patients. The development of flow cytometry technology necessitates improved capabilities for managing high-dimensional data. The traditional 8–10 colors per tube have given way to more than 50 colors when faced with contemporary high-dimensional flow cytometry data, particularly for spectral flow cytometry and mass cytometry. Conventional two-dimensional plots frequently fail to adequately represent the complex high-dimensional structures found in flow cytometry data. Aside from the perceived errors that come with manual gating analysis, it is hard to get a good read on the quantitative amount of expression of different antigens in the three-dimensional space of the target cell group [37]. Besides the restricted data processing capability, manual gating also exhibits additional disadvantages, such as reduced processing speed and vulnerability to human mistakes. It is common for different human experts to achieve varying gating outcomes for the same flow cytometry data. One of the biggest challenges to preserving clinical data quality control is this human-caused variability. Consequently, it is imperative to establish a flow cytometry analysis approach that mitigates the influence of human factors. To tackle the difficulties related to manual gating, many computational instruments have been created to automate every phase of flow cytometry data processing. These procedures include data quality assurance, normalization, visualization, cell population identification, and sample categorization. These technologies utilize a range of methods, from rule-based systems to AI models. The swift progression of AI technology and its medical applications in recent years has made this objective achievable. A variety of AI algorithms and machine learning applications for clinical flow cytometry have been developed and documented, including a wide range, from subtyping mature B-cell neoplasms and classifying acute leukemia to identifying residual illness in acute myeloid leukemia and myelodysplastic syndromes [55,56,57]. Regardless of the methods used, all of these investigations have continuously demonstrated great testing and validation efficiency. Lu et al. created and verified an AI-assisted flow cytometry methodology employing DeepFlow^TM^ software (version 2.1.1), specially tailored for the ALPS panel. The DeepFlow^TM^ program efficiently imports LMD data from the flow cytometer to the server, performs automated evaluation, and produces a detailed flow cytometry analysis report. In order to provide a preliminary diagnosis, this report is automatically prepared and includes important information, including cellular viability; immunophenotypes; cell count and proportion of T, B, and NK cells; and typical and aberrant lymphoid fractions. With the use of DeepFlow^TM^ software, cell lineages including T, B, and NK cells may be accurately clustered and differentiated. Additionally, significant subsets of immune cells, like CD8+ cytotoxic T-cell lines, CD4+ helper T-cells, CD3+ DNTs, and class-switched or non-switched B-cell lines, can be similarly distinguished. The implementation of a straightforward, fast, and automated AI-assisted system guarantees uniform, high-quality flow outcomes, resulting in precise diagnoses and improved efficiency [58].

### 2.4. AI Innovation in Immunopeptidomics and Phenotype Detection

Another illustration of AI’s classification capabilities is phenotype detection. Detection and classification of cellular phenotypes can identify the presence of specific diseases or their outcomes. The significance of molecular-level phenotype classification is particularly pronounced in specific diseases, as it directly influences prognosis and treatment strategies. The response of HIV-1 to treatment can be predicted by viral phenotype, as specific phenotypes correlate with drug resistance. Image-based phenotype detection represents an alternative method for identifying phenotypes. Image-based techniques exhibit outstanding precision at the cellular level and molecular scales. Traditional techniques for microscopic examination of tissues are labor-intensive and susceptible to subjective interpretation. Moreover, they are unable to manage the data generated from high-throughput studies. Machines are capable of matching the rapid data generation in modern medicine and can identify phenotypic changes that may be overlooked by human screening [38]. The parameters of machine testing can be modified to identify a specific phenotypic alteration or to assess a broader range of phenotypes. Image-based techniques have been utilized to assess immune responses, including macrophage activation and lymphocyte infiltration in cancer, thereby aiding in the evaluation of prognosis and treatment response. Accurate algorithms are well-established in the field of immunology for predicting the immunopeptidome. Assessing a peptide’s ability to be displayed by MHC molecules is crucial and could advance the creation of customized vaccines. Mass spectrometry (MS) is the most precise method for determining the immunopeptidome [39,59]. Nonetheless, this technique is costly and requires significant time investment. ML can identify the immunopeptidome and can be trained using existing data on the human immunopeptidome to predict the presentation of specific peptides by MHC-I molecules. In vitro studies yield data regarding peptide-MHC affinity. The data can be utilized to develop models that predict the presentability of neo-antigens by MHC molecules. Predicting epitopes for MHC-II molecules presents additional challenges due to variations in peptide length. AI algorithms demonstrate significant effectiveness in predicting MHC-II epitopes from amino acid sequences and in designing vaccines aimed at the MHC-II immunopeptidome [60,61]. Nevertheless, the accuracy of these models in predicting in vivo interactions may be compromised due to their basis in in vitro data. In addition to elucidating the human immunopeptidome, ML has contributed to vaccine development through various other mechanisms. ML can assist in the design of optimal additives for vaccines by identifying epitopes within antigens. Machines are trained using epitope properties derived from existing data on antigen epitopes. Efforts have been undertaken at the DNA and RNA levels to identify oligonucleotides with immunomodulatory properties [62]. The discussed concepts may have future applications in managing patients with immunodeficiencies, facilitating the discovery of previously unknown immunopathogenetic mechanisms and aiding in the development of more effective vaccines for this infection-prone population.

## 3. Enhancing Immunodeficiency Management: The Role of AI in Risk Stratification

The application of AI, particularly in the ML domain, to develop risk estimation systems has recently been investigated, demonstrating significant potential for aiding in the screening and diagnosis of IEI. ML investigates algorithms capable of learning from experience to uncover latent data relationships, perhaps enhancing predictions beyond conventional methods [23]. Rider et al. proposed an approach to predict infectious risk using artificial intelligence for individuals with PI. Using mathematical/probabilistic models like Bayesian networks (BNs), the aforementioned methodology integrates data on the PI’s criteria with epidemiological data [63]. BNs represent a model that integrates pertinent features, joint distributions of probability, and a coherent structure to address inquiries in the field of biomedical research [64]. BNs have been shown to be beneficial for clinical decision support in identifying lung malignancies, predicting cardiac failure, estimating survival for persons with colon cancer, assisting in liver illness diagnosis, and facilitating pathology sample evaluation, among additional applications [65,66,67,68,69,70]. Additionally, they specialize in producing predictions with less training data [71]. A BN comprises nodes that represent variables and arcs that connect these nodes to indicate causal relationships [72]. There were eight different types of IUIS disorders, each with its own set of associated feature nodes and corresponding edges: T-cell/Combined (T/CID), Predominantly Antibody (PAD), Immune Dysregulatory (PIRD), Phagocyte (PD), Innate (ID), Autoinflammatory (AID), and Complement (CD). The authors consolidated IUIS from the Expert Committee report into a single category, excluding bone marrow failing disorders and PI phenocopies. This led to the establishment of seven IUIS disorder network nodes [63]. The network model developed by Rider et al., referred to as “PI Prob”, was created by a specialist in immunology, with the parameters, specifically the conditional probabilities, derived solely from accessible EHR data. The PI Prob demonstrated effective performance on an unseen validation cohort of 75 patients with PI, encompassing a diverse array of diseases within the IUIS range. Its performance was comparable to that of other machine learning models, indicating a robust classification capability for PI patients versus control individuals and a reduced risk of excessive fitting. Besides predicting risks, PI Prob offers a prescriptive outcome to support suitable diagnostic evaluations and initiate referrals when necessary. The BN’s capacity to implement a diagnostic approach is dual-faceted. The ≥6% limit for PI Prob’s best projected risk score is intriguing since it agrees with our earlier efforts to determine a risk indicator for PI [73]. PI Prob facilitates precise and impartial risk assessment, directing users to the suitable diagnostic evaluations for patients experiencing recurring infections. Although the authors emphasize that this model demonstrated strong performance with the validation cohort, it is essential to conduct prospective testing on a larger patient population to confirm its accuracy. Two years later, the same author put out an alternative AI-based approach for stratifying PI-affected patients’ risk [74]. This study utilized claims data from a complete membership cohort of 427,110 individuals enrolled in the Texas Children’s Health Plan, starting from 1 September 2019, without any exclusions. The preliminary risk evaluation was conducted in September 2020, utilizing data from 1 September 2019 to 31 August 2020, covering the first 12 months. Subsequently, all remaining cohort members underwent iterative risk assessments at 6-month intervals for the duration of the study. Researchers employed the SPIRIT Analyzer to identify individuals at risk for PIs and IEIs [75]. The Analyzer operates by employing a predetermined weighting system for various common claim element codes, which can subsequently be enumerated to produce a PI risk assessment for a given person. The SPIRIT Analyzer is designed to evaluate 12 months of claims data, allowing for the categorization of individuals into “high”, “low-medium”, or “no claim of interest” regarding PI/IEIs. Risk stratification utilizing a tailored ML algorithm follows the SPIRIT Analyzer as the subsequent stage of the research. After 30 months of follow-up, participants who were initially deemed high risk were more likely to receive a PI/IEI-relevant diagnosis, according to the American Academy of Allergy, Asthma, and Immunology, compared to participants who were initially deemed medium-low risk or had no risk determined by SPIRIT [76]. The high-risk category constituted 1% of the original cohort (n = 297; 0.7%), with a total of 6627 individuals (2% of the total cohort) initially classified into low-, medium-, and high-risk groups. The present research investigated the application of an augmented intelligence strategy to enhance care coordination, following the initial risk triage with SPIRIT through a secondary analytical process. The goal of the second ML stage, which was intended to better categorize high-risk individuals, was to help with referral and evaluation priorities. The research indicates that patients can be ranked based on the ratio of predicted visits at risk for PI/IEIs to their total number of visits within a 12-month period. The PI Ratio serves as a tool for care coordination teams to prioritize individual evaluations according to quantified clinical urgency. Consequently, the computation of the PI Ratio offers further stratification for the purpose of directing the logistics of population care. Patients who have already been diagnosed with PI or IEI may also benefit from this pipeline’s evaluation by keeping an eye out for concerning trends, which can lead to preventive clinical care and ultimately contribute to a learning healthcare system [63]. The Phenotype Risk Score (PheRS) is a method that was established by Bastarache and colleagues. It is a method that is derived by adding up the clinical features of patients that have been weighted, with each characteristic being weighted by the inverse logarithm of its populational prevalence [77]. This approach offers advantages such as clarity, readability, and flexibility. Dr. Bastarache illustrated the effectiveness of PheRS by showing that patients with rare genetic diseases exhibit elevated risk scores. She subsequently expanded this methodology for screening to determine if patients with high-risk scores indeed possess rare pathogenic genetic variants. The group conducted an initial analysis in cystic fibrosis patients, achieving successful outcomes (Table 3) [78].

## 4. AI Improves Immunodeficiency Care: Pathogen Management

### 4.1. Anti-COVID-19 Vaccines, AI, and Immunodeficiency

More than five years have elapsed since the onset of COVID-19. There have been 771,549,718 confirmed cases of COVID-19 globally, resulting in 6,974,473 deaths. Patients with hematologic malignancies (HMs) are classified as high risk for severe COVID-19 [79]. Vaccination has reduced the overall mortality rate of HM patients to below 10%; however [80,81], patients with cancer continued to face a disproportionate burden of COVID-19 mortality compared to the general population during the Omicron variant period, as the likelihood of developing a SARS-CoV-2 vaccine-induced antibody response remained consistently lower than that of the general population [82]. These findings emphasize the necessity for ongoing initiatives and research in this domain [83]. A solid vaccine-induced antibody response is essential for safeguarding against the severity and mortality associated with the Omicron variant in immunocompromised patients [84]. Still, impaired responses to complete SARS-CoV-2 vaccination are observed in 5% to 70% of immunocompromised patients [85]. For example, it is now advised that these individuals receive a further dose [86,87]. Following immunization, the risk of SARS-CoV-2 infection is greater in HM patients due to their impaired humoral response [82]. Age, illness type, the timing of HM treatment, and the kind of HM all have a role in the overall variation in serological response rates among hematological patients. Consequently, it is highly important to understand the characteristics of HM patients who have a poor or nonexistent antibody response. This will allow for more personalized counseling about additional measures to prevent transmission, such as additional vaccine treatments, pre-exposure neutralizing SARS-CoV-2 monoclonal antibodies, and early antiviral therapy. This will help reduce the occurrence and severity of breakthrough COVID-19 in these patients. A dataset containing data on HM patients who were fully vaccinated against SARS-CoV-2 was examined by Rodríguez-Belenguer et al. Using an ML-based approach, it is possible to subdivide patients on the basis of their serological responses. The authors were able to delineate four clusters using this technique. Cluster 1 has 69.91% of patients who produce antibodies (PPGA), and it also has the largest proportion of CLL patients. Cluster 2’s small size (12 patients) makes it difficult to draw any firm conclusions, and its PPGA is the lowest at 32.2%. Cluster 3, which has a PPGA of 84.39%, is home to the vast majority of patients (71.96%). This cluster did, however, show a reduced PPGA (47.06%) among patients who underwent anti-CD 20 treatment prior to 6 months. Cluster 4 concludes with a PPGA of 66.33 percent. Patients in this cluster who also had a B-cell disease were more likely to have a weak humoral response. As the most critical variable negatively impacting antibody production, the high proportion of patients using corticosteroids likely affects the outcomes of this cluster. Utilizing this methodology in clinical practice could help pinpoint individuals who are likely to have a poor serological reaction and determine which patients could benefit from additional preventive measures [88].

### 4.2. Infections and Immunodeficiency: AI as a Diagnostic Tool

Infections can arise as a secondary complication of many non-infectious conditions. This is particularly true in cases of severe trauma, long-term use of immunosuppressants, cancer, and other chronic illnesses [89]. Infectious pathogen profiles are rarely carefully investigated in this patient group [90]. In order to help doctors promptly diagnose infections in patients with secondary immunodeficiency from causes other than HIV, Liu et al. built mathematical models using data from specific lab tests. These tests include complete blood count (CBC), C-reactive protein (CRP), procalcitonin (PCT), erythrocyte sedimentation rate (ESR), and culture results from different bodily fluids. The goal is to help doctors predict which pathogens are most likely to be present in these patients so that they can begin empirical antibiotic treatment before the culture results come back [91]. This study’s inclusion criteria are as follows: patients with hematological or solid malignancies who are on bone marrow suppression for more than two weeks as a result of chemotherapy or radiation; patients with rheumatoid autoimmune diseases who are on long-term glucocorticoids and/or cytotoxic drugs; patients with hypoproteinemia due to organ dysfunction (e.g., cirrhosis, liver failure, or protein loss from chronic kidney disease); patients on immunosuppressants following organ transplantation; patients with severe burns; patients in the intensive care unit who may have secondary immunodeficiency. The authors retained characteristics like PCT and CRP, which were strongly linked to cultivation results. This study proposes a useful predictive approach to support the empirical initiation of antibiotic treatment, based on the classification of pathogens into seven clinically relevant groups. The developed models have proven promising in guiding therapeutic decisions while awaiting microbiological results, particularly in non-specialist clinical contexts. However, the authors recognize the importance of externally validating these models to ensure their generalizability and applicability in different contexts. Future research developments will include the integration of external datasets to strengthen the reliability of the results. In summary, despite the current limitations, the study represents a concrete step toward a more timely and targeted use of empirical antibiotic therapy [91].

In this contest, the Internet of Things (IoT), particularly the Internet of Medical Things (IoMTs), has the potential to transform clinical practice in combating infectious diseases through real-time, linked, and intelligent medical diagnostics and interventions. “IoMT” refers to a network of intelligent medical devices, encompassing wearable monitors, biosensors, portable diagnostic kits, and smartphone-based readers, interconnected by wireless communication protocols like Bluetooth, Wi-Fi, and mobile networks. These technologies enable point-of-care testing (POCT), removing the necessity for traditional laboratory infrastructure by allowing for the rapid capture, processing, and transfer of clinical data directly to healthcare practitioners.

These devices generally utilize non-invasive samples and can include, but are not limited to, saliva, sweat, or urine sampling for biomarker detection of infectious diseases. This latter connectivity allows not only early diagnosis, but also some disease surveillance and outbreak containment through geolocating cases and submitting data to central health databases. Advanced biosensors in IoMT will also have the capabilities for more complex molecular assays (e.g., loop-mediated isothermal amplification [LAMP] or electrochemical detection), with or without naked-eye readout or results for a digital interface.

Some systems contain AI and ML algorithms to analyze patients’ data, to recognize patterns, and to aid clinicians in making clinical decisions. Such advancements in diagnostic tools assist in personalized treatment for an individual patient and in predicting disease prognosis and can, therefore, offer a personal approach to guide patient care, as is currently the case for immunocompromised patients [92].

## 5. The AI Method for Diagnosing Late Immunodeficiencies

As already illustrated previously, immunodeficiencies are classified into two groups: PIDs and SIDs. In the field of PIs, the genetic aspect plays a crucial role in pathogenesis: more than 500 hereditary monogenic mutations that can impact any part of the immune system and cause diseases. These congenital types exhibit a wide range of symptoms and might be present at birth. It is important to note, however, that a large proportion of PIs patients have an undetermined genetic foundation, thus complicating the identification and categorization of these diseases [93]. On the other hand, SIDs can develop at any point in life due to a wide range of chronic diseases. Malignant lymphoproliferative diseases, immunosuppressive treatments (e.g., those used following transplants or in autoimmune disorders), and chronic illnesses like malnutrition are also part of this category [94]. Each of these conditions is characterized by a non-genetic impairment of the immune system, which increases susceptibility to illness. A thorough clinical and, if possible, molecular evaluation is necessary for the management of immunodeficiencies, which can result from either congenital genetic problems or factors acquired over time. This is to guarantee that patients receive appropriate and tailored treatment [17].

### 5.1. Immunodeficiencies and Cancer Susceptibility

Neoplasms, especially hematological ones, are more likely to develop in PIs patients, which also makes them more susceptible to infections. Cancer is the second biggest killer in this group, right behind infections, and the risk of blood malignancies is much higher in people with PIs. Lymphomas account for almost two-thirds of all neoplasms discovered in individuals with PIs, making them the most common type of malignancy [95]. Some genetic variations impact both immunodeficiency and tumor formation at the molecular level, suggesting a dual role for these variations. An example is how mutations in PRKDC and other DNA repair genes can affect a patient’s immune system by promoting the growth of cancer cells and preventing B lymphocyte maturation and antibody production [96]. Similarly, activated PI3K delta syndrome (APDS) is caused by mutations in the *PI3KCD* and *PI3KR1* genes. This disorder exemplifies how immunodeficiency and cancer development overlap [97]. Mutations in the perforin-encoding *PRF1* gene are another prominent illustration of this relationship. The immune system is unable to efficiently eliminate cells that could be tumors due to these alterations, which aids in the evolution of neoplasms [97]. So, it is becoming more and more evident that immunodeficiency is associated with cancers, especially lymphomas. Immune system dysfunction is a key component in both infection susceptibility and the propensity to form neoplasms; genetic abnormalities that induce immunodeficiency can also work as oncogenic promoters, demonstrating this point.

### 5.2. Differential Diagnosis: PID vs. SID in Lymphoproliferative Disorders

Recognizing biomarkers that can distinguish PIs from secondary forms in patients with chronic B-cell lymphoproliferative disorders (B-CLPD) is of fundamental importance to improve the diagnosis, treatment, and prognosis of these patients. In an innovative study conducted by Palacios Ortega and colleagues, the authors propose that, among patients commonly classified as having SID, there may be a “hidden” subgroup of subjects with undiagnosed PID, who may present a peculiar clinical course. The aim of this observational study was to explore the use of clinical and immunological biomarkers—both traditional and more recently introduced—to support the diagnosis of PID within a cohort of patients diagnosed with SID and B-CLPD. The researchers analyzed the ability of these markers to differentiate late-onset immunodeficiencies (late-onset PID) from secondary immunodeficiencies, with the aim of refining the clinical understanding of these complex and potentially overlapping conditions. The study opens new perspectives on the possibility that some patients, apparently affected by acquired immunodeficiency, actually present a congenital defect of immunity, with important implications on the diagnostic and therapeutic level. The authors of this study identified ten immunological and clinical factors that distinguished between patients with “Suspected-PID” and those with SID. Significant differences were seen in the levels of sFLC, κ and λ, and total κ + λ among the analytical assessments. All values were significantly lower in the “Suspected-PID” group compared to the “SID” group. Analytical biomarkers have not been shown to distinguish between PID diagnoses in a significant number of cases. In the context of CVID versus SID, sFLC, and more specifically, sum κ + λ, could be a useful diagnostic for differentiating between main and secondary hypogammaglobulinemia [98]. In addition, two of the variables, “sum κ + λ” and “childhood infections”, identified PID with excellent accuracy using a tree decision model. Their AI regression approach was significant because it used other criteria as well. By combining criteria such as recurrent or severe infections in childhood with undetectable sFLC, a more consistent approach was achieved. This finding highlights the complexity of differentiating between PID and SID and emphasizes the critical need for a thorough clinical and immunological evaluation when B-CLPD is diagnosed, as it may indicate a cohort that is skewed toward hidden PID. With a positive predictive value of 100%, the tree decision model—the top predictive model for differentiating between the two groups—achieved the highest accuracy of 91.8%. Clinical criteria remain the backbone of PID diagnosis, despite the exponential growth in the number of genetic variations linked to PID etiology. Similarly, the second strongest classifier was the weight of severe or repeated infections experienced throughout childhood. Some clinical characteristics, such as the increased prevalence of autoimmune illnesses and malabsorption, are also recognized by the authors as potentially important [99] in the “Suspected PID Group”. Patients in the “Suspected-PID Group” needed more Rituximab administrations to control their malignancy and more Ig-therapy to avoid infections, and they also had higher odds of complete remission. Considered as a whole, these findings raise the possibility that these patients’ immunogenicity makes them more susceptible to immunodepletion and infection-related problems, as well as alters their clinical behavior. The discovery of a familiarity for lymphoid malignancies was both interesting and novel. Lymphoma susceptibility is associated with a large percentage of IEI-related genetic variations; the higher incidence of familial lymphoid malignancies could point to a genetic abnormality that contributes to PID and cancer. Similar to other diseases, most patients suspected of having PID did not manage to achieve B-cell reconstitution even after 10 years of treatment. This raises the possibility of an intrinsic B-cell deficiency that may have been induced by previous medications [100]. Training a regression tree decision model to identify patients with a high likelihood of late-onset PID at the time of cancer diagnosis, the AI diagnostic tool appears to appropriately target these patients. Therefore, AI could be useful for genetic studies in patients suspected of PID, enabling researchers to identify genetic abnormalities amenable to more tailored treatments. Among the 59 patients included in the “Suspected-PID Group” cohort, 66.10% had IEI-related genetic variants [50]. In addition, DNA repair pathway abnormalities were associated with nearly 20% of the variations pertaining to combination immunodeficiencies and major antibody deficits. However, it is possible that the genetic variants’ heterozygosity may be responsible for delayed cancer symptoms. This could mean that these inherited predispositions need a series of somatic mutations and environmental factors to finally show up clinically. Homozygous forms of these mutations would have probably led to earlier diagnoses of classical pediatric IEI in these cases [101].

## 6. AI in Secondary Immunodeficiency: Focus on MDS and CLL

Outside factors, such as cancer, radiation, chemotherapy, or repeated infections, can trigger SIDs. MDS and CLL are two of the most important neoplastic causes of SID in the clinic. These pathologies serve as exemplary models of malignancy-induced secondary immunodeficiency, where AI methods can provide high-resolution stratification of immunologic risk. These models facilitate clinicians to progress from static clinical staging to dynamic, data-driven methodologies for risk assessment, surveillance, and personalized intervention.

### 6.1. Secondary Immunodeficiency in MDS

MDS is characterized by immunological dysfunction caused by changes in the clonal structure of hematopoietic stem cells and an altered bone marrow microenvironment. In addition to cytopenia and inadequate hematopoiesis, this condition is distinguished by deep immunological dysregulation that affects B-cell lymphopoiesis, intracellular signalling pathways, and immune gene expression [102]. Recent research employing integrative bioinformatics analyses of CD34^+^ hematopoietic stem/progenitor cell gene expression datasets (GSE2779, GSE4619, and GSE19429) has identified significant downregulation of immune-associated genes in MDS patients, specifically RAG1, PAX5, VPREB1, CD19, and IL7R, which are implicated in B-cell development, V(D)J recombination, and antigen receptor signaling. These findings reinforce the immunodeficient phenotype of MDS, particularly in patients with low lymphoid output and frequent infections. Functional enrichment analyses conducted using the Database for Annotation, Visualisation and Integrated Discovery (DAVID) and Kyoto Encyclopaedia of Genes and Genomes (KEGG) platforms revealed that these differentially expressed genes (DEGs) are significantly enriched in pathways related to primary immunodeficiency and the hematopoietic cell lineage. Additionally, the FoxO signaling pathway has emerged as a crucial regulatory axis, particularly involving FOXO1, which integrates signals from IL-2/STAT5, TGF-β, Notch, and apoptotic pathways. Dysregulation of FOXO1 was linked to compromised lymphoid lineage commitment, altered stress responses, and advancement toward leukemic transformation. Therefore, the downregulation of FOXO1, along with associated core immune regulators such as RAG1, PAX5, and CXCR4, further implicates this transcriptional network in both immune failure and clonal evolution [103]. The use of protein–protein interaction (PPI) network construction via Search Tool for the Retrieval of Interacting Genes (STRING) and Cytoscape software (version 3.10.2) allowed for the identification of twelve hub genes with high centrality scores, many of which (e.g., PAX5, RAG1, and BACH2) are critical in adaptive immunity and are known to be inactivated or silenced in both MDS and lymphoid malignancies. Significantly, the conditional deletion of RAG1 in murine MDS models has been demonstrated to promote the transformation to AML, indicating its involvement in both immunological development and clonal hematopoietic stability [103]. Moreover, the systematic identification of downregulated immune effectors facilitates the development of ML-based classifiers capable of predicting immunological dysfunction or AML transformation based on transcriptome markers. Finally, integrating these findings into AI pipelines can support personalized risk stratification, enabling clinicians to pre-emptively identify patients with high-risk immunological profiles who may benefit from antimicrobial prophylaxis [103].

### 6.2. Secondary Immunodeficiency in CLL

CLL and its tissue counterpart, small lymphocytic lymphoma (SLL), represent mature B-cell neoplasms characterized by the progressive accumulation of immunophenotypically aberrant CD5^+^CD19^+^CD23^+^ B lymphocytes. These diseases severely compromise both cellular and humoral immunity, making patients vulnerable to repeated infections and autoimmune manifestations, which are typical characteristics of SID. Hypogammaglobulinemia indicates a deficiency in immunoglobulin synthesis resulting from neoplastic B-cell impairment and suppression of remaining polyclonal B-cells. In parallel, T-cell depletion, compromised antigen presentation, and malfunction of innate immune cells (such as NK cells and monocytes) collectively lead to reduced immunosurveillance and heightened vulnerability to opportunistic infections. CLL represents a malignancy-associated SID where both disease biology and treatment collaboratively compromise immunological integrity [104]. In this context, AI and ML are redefining CLL diagnostics and prognostics by enabling data-driven immune risk stratification. Various studies have shown that supervised ML algorithms can classify CLL using complex data from flow cytometry, gene expression profiles, DNA methylation, and blood smear images. For instance, Zhu et al. applied differential expression and network-based analysis, such as Weighted Gene Co-expression Network Analysis (WGCNA), to identify robust diagnostic biomarkers (e.g., ABCA6, PMEPA1, and EBF1) in CLL [105]. Xia et al. further established that a 26-probe DNA methylation signature could classify small B-cell lymphomas, including CLL/SLL, with excellent concordance. In addition to molecular data, deep learning techniques utilizing convolutional neural networks (CNNs) on blood smear images obtained near-perfect classification accuracy for CLL, comparable to assessments by experienced hematopathologists [106]. Flow cytometry, the diagnostic cornerstone in CLL, has been enhanced by AI models capable of predicting the necessity for extended antibody panels (e.g., CD23, FMC-7, and CD200) with real-time inference. Simonson et al. developed an ensemble model integrating CNN and RF classifiers that achieved >90% accuracy in determining when to reflexively add the CLL1 panel, thus reducing diagnostic turnaround time. These models facilitate the early detection of immunophenotypic variants and co-existing immune deficiencies [107]. Histopathologic image analysis has also benefited from CNN-based models (e.g., EfficientNetB3), though with mixed results in terms of sensitivity for nodal CLL/SLL. Alternative imaging methods, including Raman spectroscopy, have demonstrated potential. Ferè et al. demonstrated that Partial Least Squares Discriminant Analysis (PLS-DA), a multivariate statistical method, applied to Raman spectral data from peripheral smears, could distinguish CLL from healthy controls [108]. AI offers a valid means to optimize diagnosis, enabling the implementation of therapeutic strategies to prevent complications related to the state of immunodeficiency [104].

## 7. Limitations and Biases of AI in Immunodeficiency Management

Although AIs possess significant potential to revolutionize healthcare diagnosis, particularly regarding immunodeficiencies, it is essential to acknowledge their innate limitations and biases. Numerous AI models draw on data from wealthy nations, which are therefore descriptive of populations with particular demographic and epidemiological characteristics. This limitation may not adequately address the diverse clinical presentations seen in areas with limited resources or among populations exposed to endemic infections. For instance, differing patterns of fungal and mycobacterial infections in chronic granulomatous disease have been reported in Latin America compared to Europe or North America [109]. Additionally, these algorithms often depend on historical data contained in EHRs, which can accidentally reinforce existing biases associated with outdated diagnostic approaches, leading to unequal treatment [63].

LLMs have additional limitations, such as the non-reproducibility of responses, the unpredictability of generative algorithms, and a propensity to favor already known conditions over rare or recently identified diseases. As an example, due to the limited scope of their training datasets, even high-performing LLMs tested on real-world patients occasionally suggested CVID instead of more recent entities such as CTLA4 deficiency or activated PI3K delta syndrome [48].

Moreover, traditional LLMs are often impractical for use on low-cost or mobile hardware due to their massive size and computational demands. In overcoming this limitation, lightweight LLM models for edge devices have been developed (BART-base, FLAN-T5-small, and T5-small). They are smaller-scale, open-source language models that are specifically optimized to operate on low-resource hardware such as mobile devices, embedded systems, or edge computing platforms. Their reduced memory footprint and fast inference speeds make them suitable for privacy-sensitive applications, such as mental health counseling on personal devices [110].

AI approaches, in comparison to manual diagnosis by experienced immunologists and physicians, currently lack the ability to incorporate complex medical reasoning, patient context, and subtle phenotypic variations that are often critical for establishing a diagnosis in borderline or atypical cases [45]. Manual evaluation allows clinicians to synthesize longitudinal patient histories, family backgrounds, environmental exposures, and shifting symptom patterns, which organized databases typically lack. Clinical expertise is crucial for interpreting findings in the context of developing evidence-based guidelines and addressing the ethical and psychosocial aspects of patient care.

It is essential to regard AI and ML tools as complementary resources that aid clinicians in recognizing patterns in extensive datasets, identifying high-risk patients, or recommending further investigations, rather than as substitutes for human expertise [77]. Technologies must undergo rigorous validation, be continuously refined to integrate new knowledge, and be transparently incorporated into clinical procedures [19]. Careful integration of AI is essential for enhancing the standards of evidence-based, patient-centered medicine. This approach aims to improve diagnostic timeliness while protecting against inaccurate classification, missed diagnoses, and disparities in care delivery.

### Challenges of AI in Immunodeficiencies

The integration of AI into clinical practice faces significant hurdles, particularly due to physicians’ hesitance and challenges in incorporating these tools into their workflows. In the context of IEIs, the considerable phenotypic variability and complexity of these disorders mean that many clinicians lack confidence in recognizing and managing them [111]. This uncertainty can lead to two problematic extremes: either an overreliance on AI outputs without sufficient critical appraisal, or a deep skepticism that prevents adoption altogether. Compounding these issues is the “black box” nature of many AI systems, which often provide predictions without clear, interpretable reasoning. This lack of transparency conflicts with the expectations of evidence-based medicine, where clinical decisions typically require well-defined justifications [112]. Translational gaps also persist between proof-of-concept studies and scalable clinical implementation. Additionally, there remains a notable gap between promising proof-of-concept studies and robust, scalable deployment in everyday practice. While various algorithms for predicting or phenotyping primary immunodeficiencies have demonstrated retrospective success, prospective validation across diverse, real-world populations is still insufficient [113]. Future research must include extensive, multi-institutional cohorts from diverse sociodemographic contexts and illness profiles to validate usefulness, optimize thresholds, and mitigate healthcare inequities [32].

## 8. Future Perspectives and Conclusions

Primary and secondary immunodeficiencies constitute a complicated and diversified category of illnesses that significantly impact patient death and morbidity. Notwithstanding significant advancements in our comprehension of the biological and clinical foundations of these illnesses, obstacles in early diagnosis, risk assessment, and personalized care remain. The incorporation of AI and ML technology into clinical practice presents a revolutionary chance to surmount several hurdles, facilitating earlier identification, enhanced characterization, and optimized therapy options. The utilization of AI-driven tools, such as predictive models, language processing algorithms, and deep learning analyses of high-dimensional clinical and biological data, has already shown encouraging outcomes in improving diagnostic precision and aiding clinical decision making. Furthermore, the application of modern flow cytometry methods and immunopeptidomic profiling, enhanced by ML, has the potential to reveal novel biomarkers and therapeutic targets, facilitating a transition toward genuinely personalized therapy in immunology. An interdisciplinary and collaborative approach, incorporating skills from immunology, data science, bioinformatics, and ethics, will be crucial for fostering long-term innovation. Embracing technology breakthroughs and integrating them into clinical practice will render the future of immunodeficiency diagnosis and management increasingly predictive, preventative, and personalized, hence improving the quality of life for patients globally. Furthermore, the application of AI extends well beyond immunodeficiencies, with substantial advancements being made throughout the spectrum of medical research and diagnostics. Cancer researchers are making use of AI systems to sift through interactome data in search of new anticancer targets and potential therapeutic interventions. Predicting drug targets from complex, heterogeneous, and high-throughput molecular data is a breeze with machine learning-based biology analysis [114]. The integration of high-performance computing (HPC) and AI in chronic illness management enhances risk prediction accuracy, facilitating customized early treatments [115]. AI is utilized to address antimicrobial resistance by enhancing the efficacy of innovative antibiotic research and development. This includes the extraction of secondary metabolites, the evaluation of chemical libraries, and the repurposing of pharmaceuticals [116]. AI chatbots are becoming essential instruments in several medical fields, such as allergy and immunology, improving patient engagement, diagnostic precision, and individualized treatment strategies [117].

However, there are significant ethical questions raised by the broad use of these technologies. Clinical supervision and transparency are necessary when using AI in medicine. Algorithmic recommendations must be comprehensible, interpretable, and contextualized within the larger framework of medical reasoning. Inequities in healthcare delivery can also be sustained by algorithmic bias, which frequently results from non-representative training datasets and may give rise to discrimination on the basis of socioeconomic status, gender, or ethnicity. A further concern relates to patient privacy. Sensitive health data collection, sharing, and use expose patients to confidentiality violations that may have social, legal, and psychological repercussions. It is crucial to make sure that such data are processed in accordance with data protection laws, utilizing advanced cybersecurity precautions, data anonymization strategies, and privacy-preserving technologies that lower the possibility of illegal access or data breaches. AI algorithms’ lack of transparency erodes confidence and makes it more difficult to assign clinical accountability when mistakes occur. Furthermore, the question of legal liability is still largely open: it is not clear who should be held accountable for harm: the healthcare facility, the software developer, or the doctor. Given these conundrums, universal ethical and legal norms must be established in order to guarantee equity, security, and adherence to patients’ fundamental rights. AI should be used in immunodeficiency medicine to assist medical professionals in making decisions, not to replace clinical knowledge. The ability to combine clinical judgment, ethical awareness, and respect for patient rights with technological innovation will be crucial to the future of personalized medicine [118].

## Figures and Tables

**Table 1:** Summary of immunodeficiency categories, key features, and examples. This table presents an overview of the primary categories of immunodeficiencies, emphasizing their fundamental immunological defects and representative diseases. It encompasses antibody and combined immunodeficiencies; phagocyte disorders; complement deficiencies; innate immune issues; syndromic conditions; and disorders related to immune dysregulation, autoinflammation, and bone marrow failure.

Immunodeficiency Category	Key Characteristics	Representative Disorders
Antibody Deficiencies	Decreased or absent antibody synthesis, resulting in recurrent bacterial infections	Common Variable Immunodeficiency (CVID), X-linked Agammaglobulinemia (XLA)
Combined Immunodeficiencies	Deficiencies impacting both T and B lymphocytes, resulting in severe and early-onset infections	Severe Combined Immunodeficiency (SCID), DiGeorge Syndrome
Phagocyte Disorders	Reduced efficacy of phagocytes in eradicating infections	Chronic Granulomatous Disease
Complement Deficiencies	Deficiencies in the complement cascade hinder opsonization and lysis of microorganisms	C1q, C2, C3 Deficiencies
Diseases of Immune Dysregulation	Unregulated immunological activation resulting in autoimmunity and lymphoproliferation	Autoimmune Lymphoproliferative Syndrome (ALPS), IPEX Syndrome
Defects of Innate Immunity	Failures in innate immune signaling pathways	MyD88 Deficiency, IRAK-4 Deficiency
Autoinflammatory Disorders	Spontaneous inflammation resulting from dysfunction of the innate immune system and absence of autoantibodies	Familial Mediterranean Fever, TNF Receptor-Associated Periodic Syndrome (TRAPS)
Syndromic Immunodeficiencies	Immunodeficiency linked to developmental anomalies or multisystem disorders	Wiskott–Aldrich Syndrome, Ataxia–Telangiectasia
Bone Marrow Failure	Hematopoietic defects lead to cytopenias and heightened vulnerability to infections	Fanconi Anemia, Dyskeratosis Congenita, Hematologic Neoplasia

**Table 2:** AI tools and their applications in the diagnosis and management of immunodeficiencies. This table includes essential AI methodologies employed to enhance the diagnosis and management of immunodeficiencies. It encompasses prediction algorithms, electronic phenotyping, automated flow cytometry, machine learning and deep learning models, natural language processing, and tools such as the SPIRIT Analyser and phenotype capturing systems.

AI Tool/Technique	Application in Immunodeficiency
Predictive Algorithms	Early detection, risk stratification
Electronic Phenotyping (EP)	Automated illness diagnosis
Automated Flow Cytometry Analysis (the DeepFlow^TM^ software)	Detection of immune cell abnormalities
Machine Learning-Based Tool	Identification of high-risk individuals, reducing diagnostic delay
Deep Learning (DL)/Artificial Neural Networks (ANNs)	Analysis of high-dimensional data
Natural Language Processing (NLP)	Extracting information from unstructured text
SPIRIT Analyzer	Primary immunodeficiency tracking
Phenotype Capture Tool	CVID diagnosis
Regression Model	Identification of CVID patients
Logistic Regression (LR)/Elastic Nets (ENs)/Random Forests (RFs)	Estimating PIDD probability
Large Language Models (LLMs)	Assisting in diagnosis and management

**Table 3:** Overview of AI tools and their features and limitations in immunodeficiencies. Summary of key AI and ML tools used in primary immunodeficiency diagnostics, highlighting their applications, main features, and limitations.

AI Tool/Technique	Key Features	Limitations/Biases
**Predictive Algorithms**	Analyze large EHR or claims data to generate risk scores for PIDs	May depend on coding practices; lacks sensitivity to atypical phenotypes; needs validation across cohorts
**Electronic Phenotyping (EP)**	Uses computable phenotypes to identify diseases in large datasets	Portability across institutions varies; limited by data quality and completeness
**Automated Flow Cytometry (DeepFlow™)**	High-dimensional clustering, objective gating, standardized reports	Requires robust instrument data; cannot fully replace expert morphological assessment
**Machine Learning-Based Tools**	Learns hidden associations; processes complex clinical variables	Sensitive to data heterogeneity; generalizability may be limited
**Deep Learning (DL)/Artificial Neural Networks (ANNs)**	Models complex nonlinear relationships (e.g., flow data, images)	Often “black box”; interpretation challenges for clinicians
**Natural Language Processing (NLP)**	Transforms free text into structured data for ML	Depending on quality/standardization of documentation; may miss nuance
**SPIRIT Analyzer**	Uses ICD and pharmacy codes to classify patients into risk categories	Rely heavily on billing data, potentially missing under-coded presentations
**Phenotype Capture Tool**	Collects HPO-coded phenotypes; builds weighted risk scores	Expert weighting can introduce subjective bias; needs continuous update with new phenotypes
**Regression Models**	Integrate multiple phenotypes + lab data to predict risk	May overfit to local patient profiles; performance varies by healthcare system
**Logistic Regression (LR)/Elastic Nets (ENs)/Random Forests (RFs)**	Transparent, interpretable; can rank variable importance	Needs well-curated, balanced data; may miss nonlinear interactions
**Large Language Models (LLMs)**	Generate differential diagnoses, summarize complex histories	Prone to suggesting common over rare diseases; non-reproducible outputs; requires expert oversight

## Data Availability

No new data was generated for this review.
